# Seasonal Temperature and Pin Site Care Regimen Affect the Incidence of Pin Site Infection in Pediatric Supracondylar Humeral Fractures

**DOI:** 10.1155/2015/838913

**Published:** 2015-04-29

**Authors:** Hsuan-Kai Kao, Mei-Chuan Chen, Wei-Chun Lee, Wen-E Yang, Chia-Hsieh Chang

**Affiliations:** ^1^Department of Orthopedics, Chang Gung Memorial Hospital, Taoyuan County 33305, Taiwan; ^2^College of Medicine, Chang Gung University, Taoyuan County 33302, Taiwan

## Abstract

Pin site infection is a common complication after fracture fixation and bone lengthening, and daily pin site care is recommended. Weather is a strong environmental factor of infection, but few articles studied the issue of weather and pin site infection. We performed a prospective comparative study of 61 children with supracondylar humeral fractures treated by closed reduction and percutaneous pinning. The patients were divided into high-temperature season or low-temperature season by the months they received surgery. The patients within each season were further allocated to 2 groups by the different postoperative pin site care methods of daily care or noncare. The infection rate per patient was significantly higher in the high-temperature season compared to low-temperature season (45% versus 19%, *P* = 0.045). In the high-temperature season, the infection rate per patient was significantly higher in the daily care group versus the noncare group (70% versus 20%, *P* = 0.001). In the low-temperature season, the infection rate per patient was not significantly different in the daily care group versus the noncare group (10% versus 27.3%, *P* = 0.33). We recommend that careful monitoring of infection signs, rather than pin site cleaning, would be appropriate in the treatment of pediatric supracondylar humeral fractures, especially during the summer months.

## 1. Introduction

Pin site infection is a common complication after fracture fixation and bone lengthening [1–5]. The incidence has been reported as ranging from 10% to 75% [2, 4, 6–14]. Therefore, many regimens have been developed to prevent the complication [1–3, 6, 7, 15–17]. However, most of the studies have been based on individual experience or preferences rather than on well-controlled data. The great variety in patients' age, medical conditions, causes of pin fixation, and durations of fixation affects the infection rate and makes the literature difficult to use as a clinical guideline.

In addition to host and treatment factors, the environment also plays a role in infectious processes. A strong seasonal effect can be seen in many respiratory or bacterial gastrointestinal infections and in seasonally recurring childhood infections [18, 19]. It is a popular belief, but with no evidence, that weather conditions influence the incidence of pin site infection. The infection rate would be expected to be higher in hotter seasonal temperatures. Therefore, the importance of daily pin site care is emphasized more in the summer months, especially in tropical and temperate regions.

Supracondylar humeral fractures are the most common elbow fractures in children [20, 21]. The Gartland classification system is most often used to describe the severity of displacement for these fractures [22]. Type I is a nondisplaced fracture, type II is an angulated fracture with intact posterior cortex, and type III is a completely displaced fracture without cortical contact. Closed reduction and internal fixation using percutaneous Kirschner wires (K-wires) are the standard treatment for types II and III fractures [23–26]. The wires are left until fracture union, usually 4–6 weeks. The uniformity of treatment in pediatric supracondylar fracture offers a good situation for testing the effects of environmental factors and pin site care methods on the infection rate. The purpose of this prospective comparative study was to test two hypotheses: (1) pin site infection is more common in a high-temperature season and (2) daily pin care can reduce the infection rate using the model of pediatric supracondylar humeral fractures. To the best of our knowledge, this is the first study to compare the incidence of pin site infection in different seasons.

## 2. Materials and Methods

After approval by the institutional review board of the authors' hospital, children who were sent to the emergency department with a diagnosis of supracondylar humeral fracture between May 2011 and April 2013 were the study subject candidates. Inclusion criteria were skeletal immaturity and a closed supracondylar humeral fracture treated by closed reduction and percutaneous K-wire fixation. Exclusion criteria included skeletal maturity, open fractures, and fractures requiring open reduction or neurovascular exploration.

Taiwan lies on the Tropic of Cancer, and its climate is marine tropical. The entire island experiences high-temperature weather from May through to October and low-temperature weather from November through to April. We used public data on the mean monthly temperature from the Central Weather Bureau in Taiwan (http://www.cwb.gov.tw). The average temperature was 27.6°C from May through to October and 19.1°C from November through to April in the study period. Therefore, the patients were divided into 2 groups based on what the mean monthly temperature was when they received surgery. If the patients received surgery from May to October, they were allocated to the high-temperature group. If the patients received surgery from November to April, they were allocated to the low-temperature group. If the date of their operation and the date of removing their K-wires crossed the hot and cold seasons, the patient was classified by the median date between surgery and removal of the K-wires.

Pediatric trauma call duty in the authors' hospital was shared by pediatric orthopedic surgeons and orthopedic trauma surgeons. These two teams took an even number of calls throughout the study period. The study did not guide treatment decisions and operative procedures. The surgeons subjectively selected pin site care methods after the surgery. Postoperatively, the pediatric orthopedic surgeons preferred long arm casting without pin site care. The orthopedic trauma surgeons preferred thermoplastic splinting and daily cleaning of the pin sites using sterile cotton swabs impregnated with 75% alcohol. Patients organized by emergency department arrival date in this study were further allocated to two different postoperative pin site care methods, these being daily care versus noncare.

A registered nurse involved in the study instructed the parents of patients in the daily care group on how to clean the pin sites. Our pin site care regimen was as follows. (1) Clean each pin site with a 75% alcohol solution. (2) Remove crusts around pins using sterile cotton swabs. (3) Place sterile Y-type gauze on each pin site. (4) Apply sterile gauze to cover each pin. We also instructed parents in both groups on how to observe signs of infection, such as pain, redness, tenderness, foul odor, serous discharge, purulent discharge, and fever. If parents had any questions or concerns, the registered nurse provided telephone consultation.

**Figure 1:**
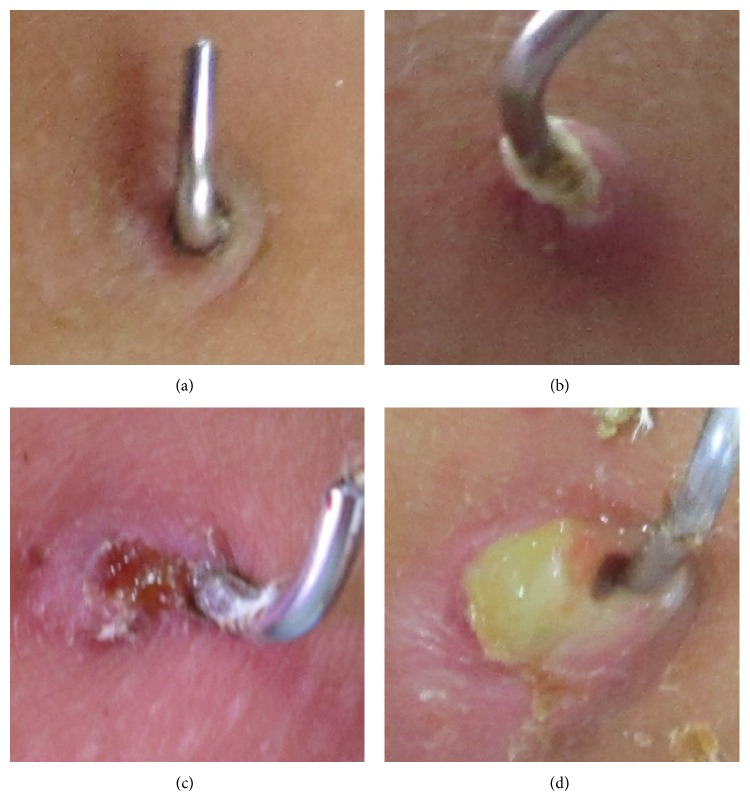
Pin site classification. (a) Normal skin. (b) Erythema without discharge. (c) Serous discharge. (d) Purulent discharge.

All patients received clinical and radiographic follow-up at 2 weeks, 4–6 weeks, and 3 months postoperatively. The cast, thermoplastic splint, and pins were removed in the clinic after 4–6 weeks. The pin sites were inspected and graded according to the system of Dahl by the same investigator during outpatient visits [6]. Grade 0 was normal skin; grade 1 was pain or erythema without discharge; grade 2 was serous discharge; grade 3 was purulent discharge; grade 4 was radiographic osteolysis; and grade 5 was ring sequestrum or osteomyelitis (Figure 1). Infection was defined as grade 2 and beyond pin site conditions. During the follow-up, any pin site that presented infection at any time was regarded as infected.

### 2.1. Statistical Analysis

Demographic data, including age, gender, body mass index (BMI, Kg/m^2^), injured side, fracture type, and number of pins, as well as the pin site care regimen and every pin site condition were recorded for all patients. The infection rate per patient and the infection rate per pin were analyzed and compared between cohorts.

A chi-square analysis or a Fisher's exact test was used where appropriate to compare categorical data between the groups. For numerical data, an independent* t*-test or the nonparametric Mann-Whitney* U* test was utilized for between-group comparisons. For each infection rate, we calculated the odds ratio (OR) and corresponding 95% confidence interval (CI). The significance level was for *P* < 0.05. Statistical analysis was performed with SPSS software (version 17.0, SPSS Inc., Chicago, Illinois).

### 2.2. Ethics Statement

The protocol for this study was reviewed and approved by the ethic committee (Institutional Review Board) of the Chang Gung Memorial Hospital in Taiwan. Parents or legal guardians of all subjects provided written informed consent, and subjects 6 years of age and older provided assent.

## 3. Results

**Figure 2:**
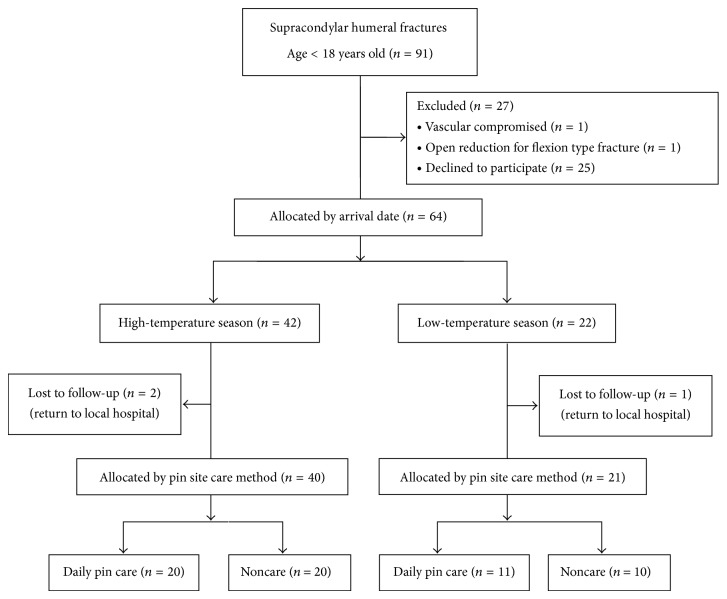
Flowchart illustrating patient enrollment, excluded patients, and distribution of treatment groups from the patient cohort.

Ninety-one children with supracondylar humeral fractures were treated by closed reduction and percutaneous K-wire fixation between May 2011 and April 2013. Of these 91 patients, 64 (70.3%) agreed to participate in the study. Of the 64 patients, three dropped out of the study because they returned to their local hospital for postoperative care. As a result, the study population consisted of 61 patients. There were 24 girls and 37 boys. All had a unilateral fracture, including 36 right elbows and 25 left elbows. The mean age at the time of the operation was 6.9 years (range 1.5 to 12.8 years). There were 9 Gartland type II fractures and 52 Gartland type III fractures. All fractures were treated in the operating room on the arrival date or the next morning. A prophylactic antibiotic with cefazolin 30 mg/kg was given within one hour before the operation. No antibiotic was prescribed after the operation. All 61 fractures were healed in 6 weeks.  Figure 2 shows a flow chart of the study population.

Forty patients had pin fixation in the high-temperature season; the other 21 patients had pin fixation in the low-temperature season. There were no significant differences in age, gender, BMI, injured side, number of pins, duration of pin retention, or pin care regimen between patients in the two seasons (Table 1). Patients in the high-temperature season were more likely to have type III fractures (*P* = 0.037). The primary outcome measure of interest was the infection rate per patient. Pin site infection occurred in 18 (45%) of 40 patients in the high-temperature season and in 4 (19%) of 21 patients in the low-temperature season. The infection rate per patient was significantly higher in the high-temperature season (*P* = 0.045).

In the high-temperature season, 20 patients were allocated to the noncare group and the other 20 patients were allocated to the daily care group. There were no significant differences in all demographic data between the two groups (Table 2). Pin site infection occurred in 4 (20%) of the 20 patients in the noncare group and in 14 (70%) of the 20 patients in the daily care group. The infection rate was significantly higher in patients who underwent daily pin site care (*P* = 0.001). The odds ratio was 9.3 (95% CI: 2.2–39.97).

In the low-temperature season, 10 patients were allocated to the noncare group and the other 11 patients were allocated to the daily care group. There were no significant differences between the two groups with regard to any of these variables (Table 3). Pin site infection occurred in 1 (10%) of the 10 patients in the noncare group and in 3 (27.3%) of the 11 patients in the daily care group. The infection rate per patient was not significantly different between these two groups (*P* = 0.331). The odds ratio was 3.3 (95% CI: 0.29–39.3).

The secondary outcome measure of interest was the infection rate per pin. Of the 144 pin sites in 61 patients, the conditions were grade 0 at 83 pin sites, grade 1 at 33 pin sites, grade 2 at 22 pin sites, and grade 3 at 6 pin sites. No grade 4 or 5 infection occurred. Infection occurred at 28 (19.4%) pin sites of 22 patients. No pin was removed before the fracture union because of infection. No local, oral, or parenteral antibiotics were prescribed during the follow-up period. All pin sites were healed without signs of infection at the 3-month follow-up visit.

Of the 144 pin sites, infection occurred at 24 (25%) of 96 pin sites in the high-temperature season and at 4 (8.3%) of 48 pin sites in the low-temperature season. In the high-temperature season, infection occurred at 6 (12.8%) pin sites in the noncare group and at 18 (36.7%) pin sites in the daily care group. In the low-temperature season, infection occurred at 1 (4.2%) pin site in the noncare group and at 3 (12.5%) pin sites in the daily pin care subgroup.  Table 4 lists the distributions of the pin site condition.

## 4. Discussion

Seasonality is characteristic of many infectious diseases. Changes in meteorological parameters have been associated with cardiovascular mortality and stroke [27–29]. The study results also support that the incidence of pin site infection is higher in hot weather. It is a routine practice to recommend cleaning the pin site to reduce the infection rate, especially in hot weather. However, this prospective comparative study had a different result. In cold weather, the infection rate was low. The patients who received daily pin care had an infection rate comparable to that of the patients who did not receive pin care. In hot weather, the infection rate was high. The patients who received daily pin care had a significantly higher infection rate than the patients who did not receive pin care.

This 2-year study of 61 patients showed a substantial summertime increase of pin site infection. These findings were consistent with the general belief and underlined the importance of more careful monitoring of infection in the summer. We also observed significant increases in the number of patients and more type III fractures in the high-temperature season. Fractures are more common in the summer months, when better weather encourages an increase in outdoor activities. Cheng et al. and Wareham et al. also reported this summertime increase and greater severity of fractures [30–32].

In our study, the infection rate was significantly higher in patients who underwent daily pin site care in the high-temperature season. There are several potential explanations. First, the dressings of pin sites in the patients who did not receive daily pin site care were applied in an aseptic manner in the operating room. Therefore the risk of infection could be reduced. Second, the families were not medical personnel, and the quality of pin site care could not be guaranteed. Third, our pin site care regimen was not optimal in preventing infection.

The optimal method of pin site care is still controversial. Many regimens have been developed to diminish the pin site infection, with variable success rates [1, 2, 6, 7, 15–17]. A variety of regimens, including soap, saline, hydrogen peroxide, alcohol, and an alcohol solution of chlorhexidine or nothing, have been reported based on individual preferences rather than a well-controlled comparative study [2, 3, 6, 17]. A recent Cochrane review demonstrated that there was insufficient evidence to be able to identify a strategy that minimizes infection rates [33].

The frequency of pin site care is also a controversial issue in clinical practice. W-Dahl et al. evaluated 50 patients with external fixation. They found no difference between daily and weekly pin site care with regard to the severity of infections and frequency of infection rate [8]. Camathias et al. also reported no difference in soft tissue interface, pin stability, and radiographic osteolysis between the daily pin site care group and the noncare group. They concluded that routine pin site care in external fixation is unnecessary [34]. We conducted a prospective study and controlled disease, location, operation, age, and duration of pin retention to validate the therapeutic efficacy of two different pin site care protocols in different temperatures. The results did not support daily pin site care in preventing infection in pediatric supracondylar humeral fractures.

Most classification systems of pin site infections are based on clinical signs rather than on microbiological features to grade the level of pin site infection [35–37]. A standard classification system has not been established yet. In this study, we defined infection as Dahl's grade 2 and beyond pin site conditions. Grade 1 was skin erythema without discharge. This could be caused by a local inflammatory reaction during the normal healing process and confound the results. By a critical definition of Dahl's grade 2 infection and beyond, the overall infection rate by patients was 36.1% and the infection rate by pins was 19.4%.

There are several limitations of this study. First, wound cultures were not performed in general practice, and microbiological data could not be obtained to confirm the infection. Second, potential non-weather-related factors, such as patient compliance with pin site care at home and the occurrence of more Gartland type III fractures in summer, could affect the result. Third, the evaluators were not blinded to the pin site care regimens. It potentially could affect the classification of infection. Fourth, the study was conducted in the model of pediatric supracondylar humeral fractures. The results may be different in elderly patients or in pinning of the lower extremities.

## 5. Conclusions

The rate of pin site infection was significantly higher in the high-temperature season, and careful monitoring of infection signs can be recommended. The prospective comparative study did not support daily pain site care in preventing infection, especially in the high-temperature season.

## Figures and Tables

**Table 1:** Patient data.

Variable	High-temperature season	Low-temperature season	*P* value
Number of patients^*^	40	21	
Mean age (years)^+^	7 ± 2.6	6.7 ± 2.6	0.66
Male/female^*^	27/13	10/11	0.13
BMI (Kg/m^2^)^+^	17.2 ± 3.7	15.8 ± 2.4	0.11
Right/left^*^	23/17	13/8	0.74
Fracture type II/III^*^	3/37	6/15	0.037
Average number of pins	2.4	2.3	0.42
Pin retention (days)^+^	34.4 ± 8	31.3 ± 5.3	0.13
Noncare/daily care^*^	20/20	10/11	0.86
Infected patients^‡^	18 (45)	4 (19)	0.045

^*^The values are given as the number of patients.

^+^The values are given as the mean and standard deviation.

^‡^Data are number (%) of patients.

*P* < 0.05 is considered statistically significant.

**Table 2:** Patient data in the high-temperature season.

Variable	Noncare group	Daily care group	*P* value
Number of patients^*^	20	20	
Mean age (years)^+^	6.9 ± 2.1	7.1 ± 3	0.85
Male/female^*^	13/7	14/6	0.74
BMI (Kg/m^2^)^+^	17.6 ± 3.3	16.9 ± 4	0.55
Right/left^*^	11/9	12/8	0.75
Fracture type II/III^*^	1/19	2/18	1.00
Average number of pins	2.35	2.45	0.57
Pin retention (days)^+^	33.4 ± 5	35.4 ± 10	0.45
Infected patients^‡^	4 (20)	14 (70)	0.001

^*^The values are given as the number of patients.

^+^The values are given as the mean and standard deviation.

^‡^Data are number (%) of patients.

*P* < 0.05 is considered statistically significant.

**Table 3:** Patient data in the low-temperature season.

Variable	Noncare group	Daily care group	*P* value
Number of patients^*^	10	11	
Mean age (years)^+^	5.9 ± 2.3	7.5 ± 2.6	0.16
Male/female^*^	4/6	6/5	0.41
BMI (Kg/m^2^)^+^	16.1 ± 2.4	15.5 ± 2.3	0.57
Right/left^*^	6/4	7/4	0.61
Fracture type II/III^*^	3/7	3/8	0.63
Average number of pins	2.4	2.2	0.29
Pin retention (days)^+^	31.4 ± 4.4	31.3 ± 6.1	0.96
Infected patients^‡^	1 (10)	3 (27.3)	0.33

^*^The values are given as the number of patients.

^+^The values are given as the mean and standard deviation.

^‡^Data are number (%) of patients.

*P* < 0.05 is considered statistically significant.

**Table 4:** The distribution of pin site condition.

	High-temperature season		Low-temperature season	
	Noncare group	Daily care group	Noncare group	Daily care group
Grade 0	35 (74.5)	19 (38.8)	17 (70.8)	12 (50)
Grade 1	6 (12.8)	12 (24.5)	6 (25)	9 (37.5)
Grade 2	4 (8.5)	14 (28.6)	1 (4.2)	3 (12.5)
Grade 3	2 (4.3)	4 (8.2)	0	0
Total	**47 (100)**	**49 (100)**	**24 (100)**	**24 (100)**

Data are number (%) of pins.

Because of rounding, percentages may not add to 100%.
